# Does implementing a rapid response system decrease the number of in-hospital cardiac arrests?

**DOI:** 10.1186/cc9896

**Published:** 2011-03-11

**Authors:** R So, L Te Velde, H Ponssen, M Frank, S Hendriks, E Oskam

**Affiliations:** 1 Albert Schweitzer Hospital, Rotterdam, the Netherlands

## Introduction

Resulting from the Dutch VMS Safety Program 'Prevent Injury, Work Safely', we recently started to implement a rapid response system (RRS) in our hospital. The purpose of the RRS is to recognize and treat the patients with clinical warning signs early on the ward to reduce preventable hospital-wide avoidable injury. We present the first outcome data for the implementation of the RRS.

## Methods

From 1 May 2008 to 1 May 2009 we implemented in both clinical locations of our hospital a RRS, which has three basic limbs: an afferent limb (RRS activation card), a physician-led medical emergency team (MET) and an evaluation/feedback limb. We collected data regarding all MET calls from 1 May 2008 to 1 July 2010 and we focused on the number of in-hospital cardiac arrests (CA).

## Results

See Table 1.

**Table 1:** Number per 1,000 discharged patients

	2007	2008	2009	2010
MET calls				
Dordrecht	0	1.2	3.2	2.9
Zwijndrecht	0	6.4	11.8	10.9
In-hospital CA				
Dordrecht	1.4	1.2	1.4	1.4
Zwijndrecht	2.6	1.3	0.6	0.6

## Conclusions

Implementation of a RRS can decrease the number of in-hospital cardiac arrests dramatically and thus avoid (serious) adverse events and possible deaths. Possible success factors include: timely activation of the RRS, the degree of implementation of the RSS, and timely agreed restrictive measurements on the general ward.

